# Comparative mechanisms for O_2_ storage and metabolism in two Florida diving birds: the anhinga (*Anhinga anhinga*) and the double-crested cormorant (*Nannopterum auritum*)

**DOI:** 10.1007/s00360-024-01593-x

**Published:** 2024-12-20

**Authors:** Jeff White, Elizabeth R. Schell, Neal J. Dawson, Kevin G. McCracken

**Affiliations:** 1 Department of Biology, University of Miami, Coral Gables, FL USA; 2 Department of Public and Ecosystem Health, Cornell University College of Veterinary Medicine, Ithaca, NY USA; 3 School of Biodiversity, One Health and Veterinary Medicine, University of Glasgow, Glasgow, UK; 4 Department of Marine Biology and Ecology, Rosenstiel School of Marine, Atmospheric, and Earth Science, University of Miami, Miami, FL USA; 5 Human Genetics and Genomics, University of Miami Miller School of Medicine, Miami, FL USA

**Keywords:** Breath-hold diving, Hemoglobin, Muscle metabolism, Myoglobin, Oxygen storage

## Abstract

Air-breathing vertebrates face many physiological challenges while breath-hold diving. In particular, they must endure intermittent periods of declining oxygen (O_2_) stores, as well as the need to rapidly replenish depleted O_2_ at the surface prior to their next dive. While many species show adaptive increases in the O_2_ storage capacity of the blood or muscles, others increase the oxidative capacity of the muscles through changes in mitochondrial arrangement, abundance, or remodeling of key metabolic pathways. Here, we assess the diving phenotypes of two sympatric diving birds: the anhinga (*Anhinga anhinga*) and the double-crested cormorant (*Nannopterum auritum*). In each, we measured blood- and muscle-O_2_ storage capacity, as well as phenotypic characteristics such as muscle fiber composition, capillarity, and mitochondrial arrangement and abundance in the primary flight (pectoralis) and swimming (gastrocnemius) muscles. Finally, we compared the maximal activities of 10 key enzymes in the pectoralis, gastrocnemius, and left ventricle of the heart to assess tissue level oxidative capacity and fuel use. Our results indicate that both species utilize enhanced muscle-O_2_ stores over blood-O_2_. This is most apparent in the large difference in available myoglobin in the gastrocnemius between the two species. Oxidative capacity varied significantly between the flight and swimming muscles and between the two species. However, both species showed lower oxidative capacity than expected compared to other diving birds. In particular, the anhinga exhibits a unique diving phenotype with a slightly higher reliance on glycolysis and lower aerobic ATP generation than double-crested cormorants.

**Supplementary Information:**

The online version contains supplementary material available at 10.1007/s00360-024-01593-x.

## Introduction

Air-breathing vertebrates face many physiological challenges while breath-hold diving. In particular, they must endure intermittent periods of declining oxygen (O_2_) stores without the ability to replenish O_2_ until surfacing. Therefore, diving activity and expenditure of O_2_ stores must be balanced by efficient O_2_ recovery periods at the surface (Burggren et al. 2024). The highly energetic foraging behavior exhibited by many diving species requires a reliable supply of O_2_ to power the locomotory muscles without fatigue. Previous studies have shown that most divers remain within their aerobic dive limit and therefore maintain aerobic metabolism while diving (Kooyman et al. 1998; Ponganis et al. 1997a; 1997b). Yet as a dive progresses, O_2_ stores may become depleted while muscle activity and metabolism continue to demand a steady supply of O_2_. This potential mismatch between the O_2_ supply and demand from the tissues has driven the evolution of multiple strategies to increase the efficiency of O_2_ delivery and use across diving taxa (reviewed by Ponganis 2015).

### Diving in the Suliformes

The bulk of research into the diving physiology of birds has occurred on penguins (Culik et al. 1996; Kooyman and Ponganis 1998; Ponganis et al. 1997a, 1999), alcids (Davis and Guderley 1990; Kovacs and Meyers 2000; Elliott et al. 2010), and ducks (Turner and Butler 1988; Bevan and Butler 1992; Dawson et al. 2016; Schell et al. 2023, 2024a, 2024b). However, the physiological mechanisms of O_2_ storage and use in other diving birds, such as the Procellariiformes (Dunphy et al. 2015) and the Suliformes, have been less well studied. The order Suliformes contains three diving families: Sulidae (gannets and boobies), Phalacrocoracidae (cormorants and shags), and Anhingidae (darters). The Suliformes exhibit a wide range of diving behaviors from pelagic plunge dives to shallow water stealth hunting. The deepest divers of the order are the blue-eyed shag species (*Leucocarbo* spp.) of the Southern Ocean, which may dive to depths > 35 m with durations over 120 s (Croxall et al. 1991; Wanless et al. 1992). However, most Suliformes species are shallow to mid-depth divers remaining within the top 30 m of the water column (Hennemann 1985; Wilson and Wilson 1988; Adams and Walter 1993; Ryan 2007; Ropert-Coudert et al. 2009). While studies have explored topics such as the physiological consequences of plunge diving in Australasian gannets (*Morus serrator*; Green et al. 2009) and the diving response and cardiac performance of cormorants (Schmid et al. 1995; Watanuki et al. 1996; Enstipp 1999; Grémillet and Wilson 1999; Enstipp et al. 2001; Watanabe et al. 2011), there has been comparatively little work on other physiological mechanisms in these species.

Here, we assess the phenotypes of two sympatric diving Suliformes: the anhinga (*Anhinga anhinga*) and the double-crested cormorant (*Nannopterum auritum*). These two species exhibit drastically different morphology and diving behavior (Owre 1967; Hennemann 1983, 1985). While the double-crested cormorant is an energetic pursuit diver, the anhinga presents an extremely uncommon, passive diving behavior, frequently employing a lie-in-wait stealth tactic. Like all cormorants and darters, both study species have wettable plumage which reduces buoyancy, and in the case of the anhinga, directly impacts metabolic rate (Henneman 1985). Previous studies have compared the foraging ecology of sympatric cormorants and darters (Owre 1967; Hennemann 1983, 1985; Ryan 2007), but none have assessed the underlying physiological mechanisms contributing to these different foraging behaviors. Therefore, we aimed to compare common traits associated with diving capacity to determine if the unique morphology and behavior of the anhinga was associated with modifications to their underlying physiology. We expected that the deeper, more energetic diving behavior of the double-crested cormorant would be supported by a higher O_2_ storage capacity and a more oxidative phenotype, similar to the sea ducks presented by Schell et al. (2023, 2024a, 2024b). In both species, we measured blood- and muscle-O_2_ storage capacity, as well as phenotypic characteristics such as muscle fiber composition, capillarity, and mitochondrial localization and abundance in the primary flight (pectoralis) and swimming (gastrocnemius) muscles. Finally, we compared the maximal activity of 10 key enzymes in the pectoralis, gastrocnemius, and left ventricle to assess tissue level oxidative capacity and fuel use.

## Methods

### Sample collection

Field studies occurred between December 2021 and March 2022 prior to the breeding season in Florida. We collected adult anhingas (*n* = 10) and double-crested cormorants (*n* = 11) from lakes and drainage ditches at a rock quarry site in Charlotte County, Florida, and one additional double-crested cormorant from an aquaculture facility in Lee County, Florida (Online Resource 1). The breadth of muscle samples required for analysis necessitated the harvesting of these individuals. All birds were collected by trained personnel using a 12-gauge shotgun (no. 2 or 3 non-toxic shot) and, when necessary, euthanized by cervical dislocation following AVMA Guidelines for the Euthanasia of Animals 2021 (AVMA, 2021). All methods were approved by state and federal agencies and by the University of Miami Institutional Animal Care and Use Committee (IACUC).

Upon collection, we immediately extracted whole arterial blood via cardiac puncture of the ventricles using 3 mL syringes rinsed and flushed with 0.5 M EDTA (pH 8.0) as an anticoagulant. The heart, left and right pectoralis, gastrocnemius, and lungs were removed and weighed prior to sample collection to determine each tissue’s percentage of the total body mass. We then dissected muscle samples from an intermediate depth (50% muscle depth) of the pectoralis and the gastrocnemius following Dawson et al. (2016, 2020). Samples of each muscle along with samples cut from the left ventricle were then flash frozen in liquid nitrogen (N_2_) to be used for myoglobin concentration ([Mb]) and enzyme assays. A small segment of the gastrocnemius was then fixed at resting length in 2% glutaraldehyde with sodium cacodylate buffer for use in transmission electron microscopy (TEM) at the Canadian Centre for Electron Microscopy at McMaster University in Hamilton, Ontario, Canada. Then, additional samples from both the gastrocnemius and the pectoralis were cut transversely, affixed to cork in an embedding medium, flash frozen with liquid N_2_-cooled isopentane, and stored at − 80 °C for histochemistry analysis.

### Muscle histochemistry

Once in the lab, the frozen gastrocnemius and pectoralis samples were sectioned transverse to the fiber length in a − 20 °C Leica Cryostat (Leica Biosystems, Buffalo Grove, IL, USA) to a thickness of 20 μm. These sections were stained for succinate dehydrogenase activity (Deveci et al. 2001; Scott et al. 2009), imaged with light microscopy, and analyzed using the FIJI software (Schindelin et al. 2012) to determine oxidative and glycolytic fiber compositions. We then quantified the number and density of capillaries and the capillary-to-fiber ratio for both fiber types in the gastrocnemius.

The glutaraldehyde fixed sections of the gastrocnemius (*n* = 6 per species) were post-fixed in buffered OsO_4_ (1%) for 1 h, dehydrated in ethanol, and finally embedded in epoxy resin following Scott et al. (2009). Next 0.5 μm transverse sections were stained with toluidine blue from which 80 nm sections were stained with uranyl acetate and lead citrate. These sections were then imaged via TEM. We then analyzed 10 images per individual to reach a stable mean and to account for heterogeneity. We classified the mitochondria by location within the cell. Subsarcolemmal mitochondria were defined as lying between the cell membrane and the peripheral myofibrils, whereas intermyofibrillar mitochondria were within the myofibrils. The images were analyzed in FIJI (Schindelin et al. 2012) to determine the proportion of mitochondria in each position in both oxidative and glycolytic fibers.

### Enzymatic assays

We next assayed the maximal activities of 10 metabolic enzymes following Dawson et al. (2016, 2020) and Schell et al. (2023) in the laboratory at the University of Glasgow (Glasgow, Scotland, UK). These assays were performed concurrently using the same reagents and protocols as Schell et al.’s (2023) samples of North American waterfowl. These enzymes included markers of glycolytic ATP production at the beginning (hexokinase; HK) and end (pyruvate kinase; PK) steps of glycolysis as well as lactate dehydrogenase (LDH), a key catalyst for the interconversion of pyruvate to lactate. The enzyme 3-hydroxyacyl-CoA dehydrogenase (HOAD) is an important catalyst in the break-down of fatty acids during β-oxidation and was used to provide an estimate of lipid oxidation capacity. Citrate synthase (CS), an enzyme in the tricarboxcylic acid (TCA) cycle, was used as a marker for mitochondrial volume density. We also included multiple key enzymes of the electron transport chain (ETC) including succinate dehydrogenase (SDH), corresponding to Complex II and part of the TCA cycle; cytochrome c oxidase (COX), corresponding to Complex IV and the terminal O_2_ consumer; and ATP synthase (ATPSyn), corresponding to Complex V and the final step of oxidative phosphorylation where ATP is generated. Finally, we measured the activities of adenylate kinase (AK) and creatine kinase (CK), which are involved in substrate level phosphorylation and the transfer of high-energy phosphates.

For these assays, the frozen samples of pectoralis (*n* = 19), gastrocnemius (*n* = 20), and left ventricle (*n* = 20) were homogenized in 20 volumes of ice-cold homogenization buffer (100 mM potassium phosphate, 1 mM EGTA, 1 mM EDTA, 0.1% Triton X-100; pH 7.2) and centrifuged for 2 min at 2,000 rpm. The resulting supernatant was collected, and assays were conducted at avian body temperature (41 °C) using substrate concentrations previously found to be saturating (Dawson et al. 2016). We ran all assays in triplicate, and the change in absorbance was measured using a Spectramax Plus 384 spectrophotometer (Molecular Devices, Sunnyvale, CA, USA). The maximal activity for each enzyme was calculated as the difference between the reaction rate with all substrates present minus the background reaction rate (the rate in the presence of an inhibitor or without the key substrate) and is reported as units of micromole substrate per gram of tissue per minute (µmol g^–1^ tissue min^–1^ hereafter U g^−1^). All enzyme assays were run concurrently with duck enzyme assays reported previously by Schell et al. (2023).

### Blood- and muscle-O_2_ storage capacity

Immediately following collection, we measured [Hb] in the blood via cardiac puncture using a HemoCue 201+ Analyzer (HemoCue America, Brea, CA, USA). This chemically converts Hb to azidemethemoglobin and measures the absorbance at 570 and 880 nm (Hudson-Thomas et al. 1994). We then adjusted the measured values by −1 mg dL^–1^ following published avian [Hb] correction factors (Simmons and Lill 2006; Qualls et al. 2017). We next measured the Hct in quadruplicate using heparinized 75 mm capillary tubes spun for 5 min in a ZIPocrit centrifuge (LW Scientific, Lawrenceville, GA, USA). Finally, mean corpuscular hemoglobin concentration (MCHC) was calculated to determine the average [Hb] in red blood cells using the following equation (Campbell and Ellis 2013):

$$MCHC = {{\left( {Hb\left[ {g\,dL^{{ - 1}} } \right] \times 100} \right)} \mathord{\left/ {\vphantom {{\left( {Hb\left[ {g\,dL^{{ - 1}} } \right] \times 100} \right)} {\left( {Hct\left[ \% \right]} \right)}}} \right. \kern-\nulldelimiterspace} {\left( {Hct\left[ \% \right]} \right)}}$$

We determined the [Mb] in the pectoralis, gastrocnemius, and left ventricle following a modified Reynafarje (1963) method as used by Dawson et al. (2016, 2020). We first homogenized frozen tissue in 19.25 volumes (1 mL buffer per 1 g of tissue) of ice-cold homogenization buffer (40 mM potassium phosphate; pH 6.6) and centrifuged for 99 min at 13,700x g at 4 °C (Dawson et al. 2016). We then transferred the supernatant to a 25 mL boiling flask under constant rotation at 100 rpm and exposed it to pure carbon monoxide (CO) for 8 min. We then added sodium dithionite and continued CO exposure for an additional 2 min to ensure the complete reduction of Mb. We then diluted the samples 19.5x with a homogenization buffer prior to transferring to a 1 mL cuvette (10 mm optical path length). We measured the optical density of each sample in triplicate at 538 and 568 nm against a homogenization buffer blank on a VWR V1200 Spectrophotometer (VWR, Radnor, PA, USA). Following Dawson et al. (2016), we determined the [Mb] (mg g^–1^) using the following equation:

$$\left[ {Mb} \right] = \left( {OD_{{538}} - OD_{{568}} } \right) \times 112.6$$

Once fully excised, we compared the mass of the locomotory muscles, heart, and lungs in both species. We then calculated the proportion of the total body mass composed of each of these tissues. From this we estimated the total amount of Mb available in each muscle as a source for O_2_.

### Statistical analyses

We performed Welch’s two sample t-tests or Kruskal-Wallis tests for most comparisons between the two species. We assessed [Mb] and enzyme maximal activity among the three tissue types collected (i.e., pectoralis, gastrocnemius, and left ventricle) by performing analysis of variance (ANOVA). Then for enzyme maximal activity, we performed principal component analysis (PCA) to determine the relationships between each of the measured enzymes and the two species. All analyses were performed using R Statistical Software (R Core Team, 2022) in RStudio (RStudio Team, 2023). A full table of the statistical results is given in Online Resources 2 and 5.

## Results

### Muscle phenotype between species

**Fig. 1:**
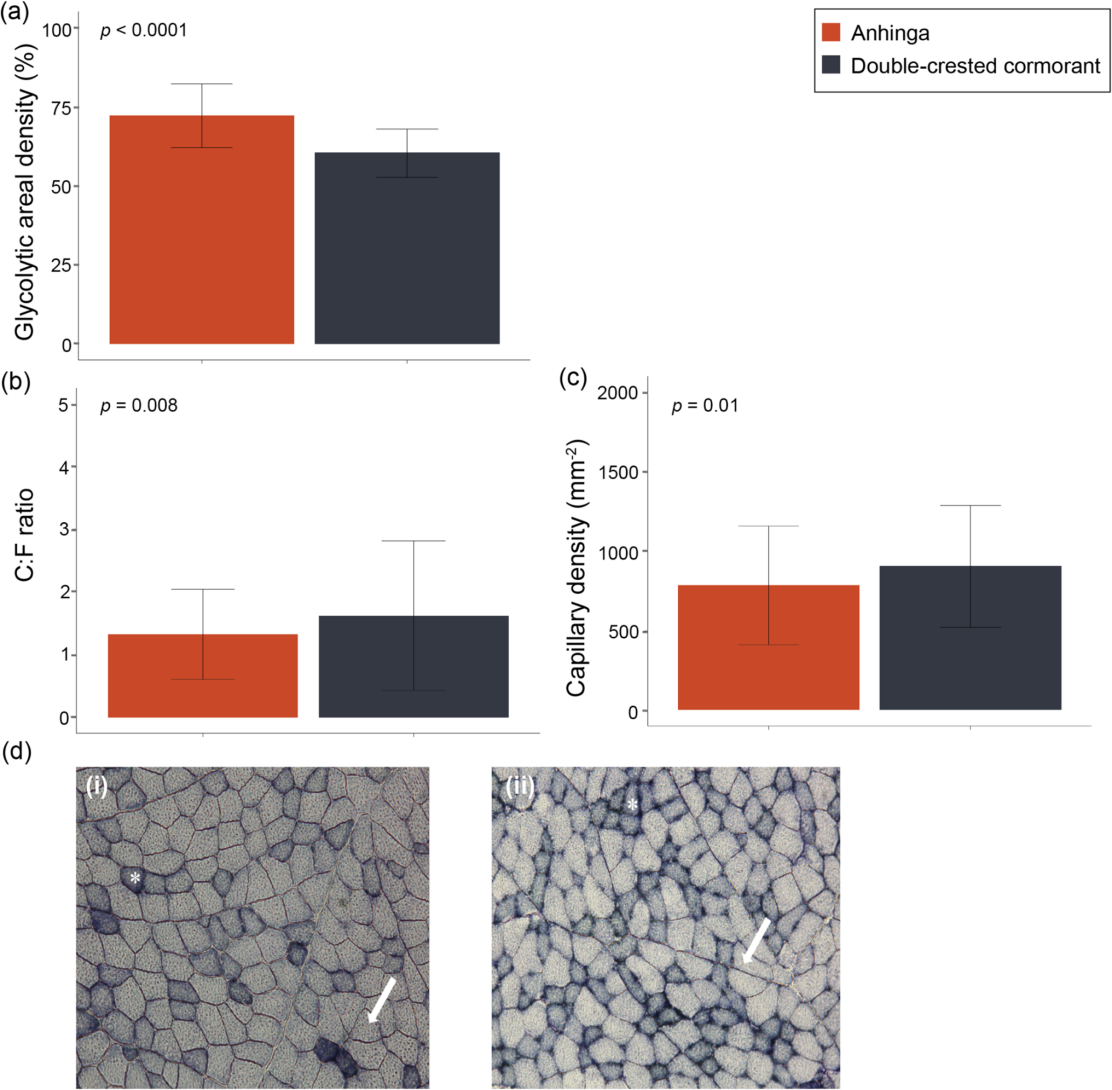
Comparison of gastrocnemius **a** glycolytic areal density (%), **b** capillary/fiber ratio, and **c** capillary density (mm^–2^) of anhingas and double-crested cormorants. Bars show the median and standard deviation of the data recorded from multiple measures of the same 10 individuals of each species. Significance levels were derived from t-tests or Kruskal-Wallis tests with the alpha value set as *p* < 0.05. **d** Succinate dehydrogenase staining of gastrocnemius muscle in (i) anhinga and (ii) double-crested cormorant showing the areal density of oxidative (asterisk) and glycolytic (arrow) fibers

While there was no difference in the transverse fiber area between species, glycolytic fibers were 1.8–1.9x larger than oxidative fibers in both species (*p* = 0.003). The anhinga had a significantly higher areal density of glycolytic fibers in both the pectoralis and the gastrocnemius (*p* = 2.2 × 10^–16^; Fig. 1a; Table 1; Online Resource 2). Additionally, the double-crested cormorant had a higher capillary density (*p =* 0.01) and capillary-to-fiber ratio (*p* = 0.008) in the gastrocnemius than the anhinga (Fig. 1b-c; Table 1).

**Table 1:** Mean and standard deviation (SD) of muscle traits compared between the anhinga (*Anhinga anhinga*) and double-crested cormorant (*Nannopterum auritum*). Results of full statistical analyses are given in Online Resource 2. Significances were derived from t-tests or Kruskal-Wallis tests with the alpha value set as *p* < 0.05. sample sizes for anhinga (*n* = 10) and double-crested cormorant (*n* = 10) unless otherwise noted by superscript and corresponding footnote

Trait	Anhinga	Double-crested cormorant
Body mass (g)^3^	1445 ± 121	2304 ± 467^***^
*Gastrocnemius*		
Mass (g)	7.3 ± 1.7	31.6 ± 7.1^***^
Percent total mass (%)	0.5 ± 0.1	1.4 ± 0.1^***^
Glycolytic fiber areal density (%)	72.2 ± 10.1^***^	60.4 ± 7.6
Oxidative fiber areal density (%)	27.8 ± 10.1	39.6 ± 7.6^***^
Glycolytic fiber mitochondrial volume density^4^	0.12 ± 0.02	0.17 ± 0.04^*^
-- Subsarcolemmal mitochondrial volume density^4^	0.03 ± 0.01	0.05 ± 0.02^*^
-- Intermyofibrillar mitochondrial volume density^4^	0.09 ± 0.01	0.11 ± 0.02
-- Proportion subsarcolemmal^4^	0.26 ± 0.03	0.32 ± 0.03^**^
Oxidative fiber mitochondrial volume density^4^	0.29 ± 0.06	0.33 ± 0.02
-- Subsarcolemmal mitochondrial volume density^4^	0.11 ± 0.04	0.13 ± 0.01
-- Intermyofibrillar mitochondrial volume density^4^	0.18 ± 0.02	0.21 ± 0.02
-- Proportion subsarcolemmal^4^	0.38 ± 0.06	0.38 ± 0.03
Average mitochondrial volume density^4^	0.21 ± 0.10	0.25 ± 0.09
Glycolytic fiber transverse area (µm^2^)	2706.36 ± 646.91	2465.57 ± 562.74
Oxidative fiber transverse area (µm^2^)	1389.09 ± 446.14	1377.44 ± 288.41
Capillary density (mm^–2^)	738.35 ± 371.97	905.52 ± 367.79^*^
Capillary/fiber ratio	1.33 ± 0.58	1.62 ± 0.45^**^
*Pectoralis*		
Mass (g) (% total)	165.4 ± 12.4	227.4 ± 46.1^***^
Percent total mass (%)	11.8 ± 0.6^***^	9.9 ± 0.7
Glycolytic fiber areal density (%)	2.7 ± 2.4^***^	0.00 ± 0.2
Oxidative fiber areal density (%)	97.3 ± 2.4	100.0 ± 0.2^***^
*Heart*		
Mass (g) (% total)	10.7 ± 1.3	18.5 ± 1.1
Percent total mass (%)	0.8 ± 0.04	0.8 ± 0.06
*Lungs*		
Lung mass (g)	10.7 ± 2.3	25.8 ± 6.6^**^
Percent total mass (%)	0.8 ± 0.1	1.1 ± 0.3^***^

*p < 0.05, **p < 0.001; ***p < 0.0001; ^3^cormorant: *n* = 12; ^4^both species *n* = 6

**Fig. 2:**
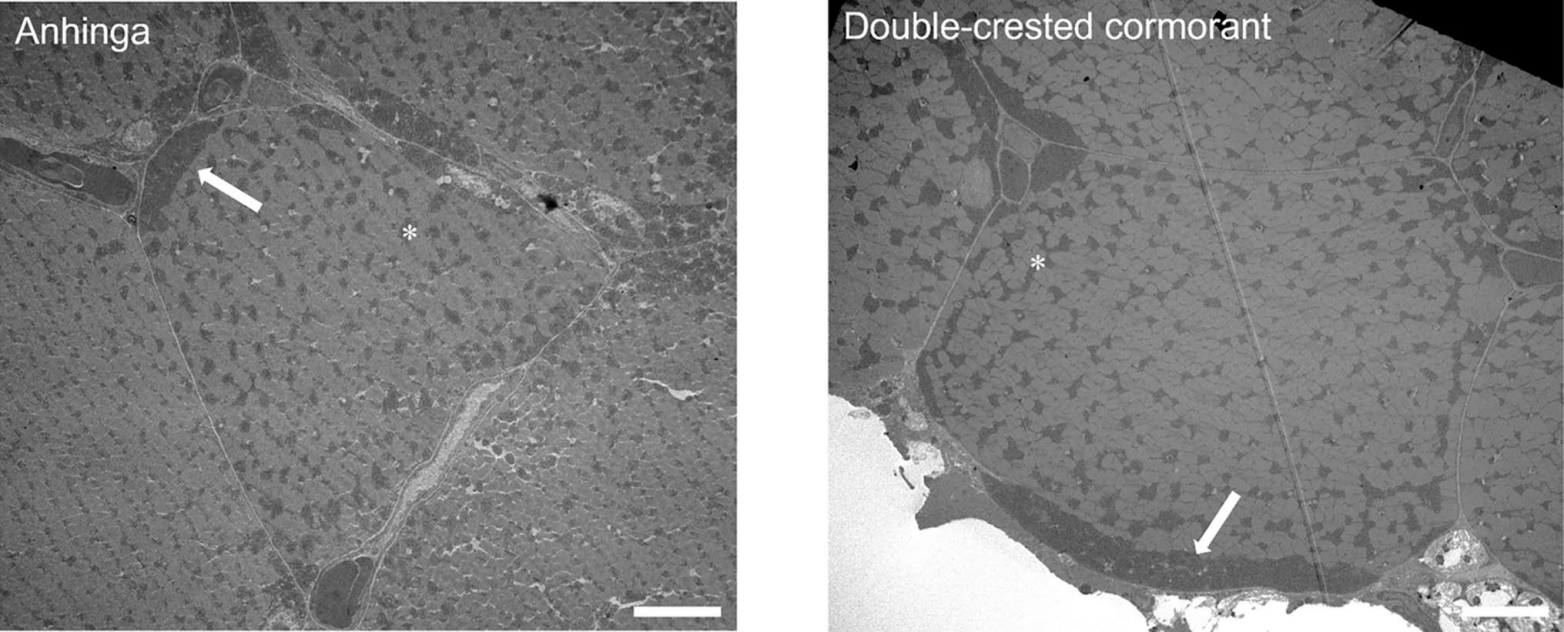
Comparison of mitochondrial volume density of anhingas and double-crested cormorants. Representative TEM images of an oxidative fiber from an anhinga (left) and a double-crested cormorant (right) depicting subsarcolemmal (arrow) and intermyofibrillar (asterisk) mitochondria. Scale bar 8 μm. There were no differences in overall mitochondrial volume density or subfractions in the oxidative fibers. However, in the glycolytic fibers a higher overall mitochondrial volume density in the double-crested cormorants can be attributed to a larger subsarcolemmal subfraction

We next assessed the overall mitochondrial volume density in the gastrocnemius and found no differences in oxidative fibers. However, double-crested cormorant glycolytic mitochondrial density was higher than in anhingas (*p* = 0.04; Table 1; Online Resource 2), driven by an increase in the volume density of subsarcolemmal mitochondria in these fibers (*p* = 0.02). The proportion of the total mitochondrial volume composed of the subsarcolemmal subfraction was significantly higher in the double-crested cormorant glycolytic fibers (*p =* 0.007; Fig. 2; Table 1).

### Enzyme activity in the pectoralis

**Fig. 3:**
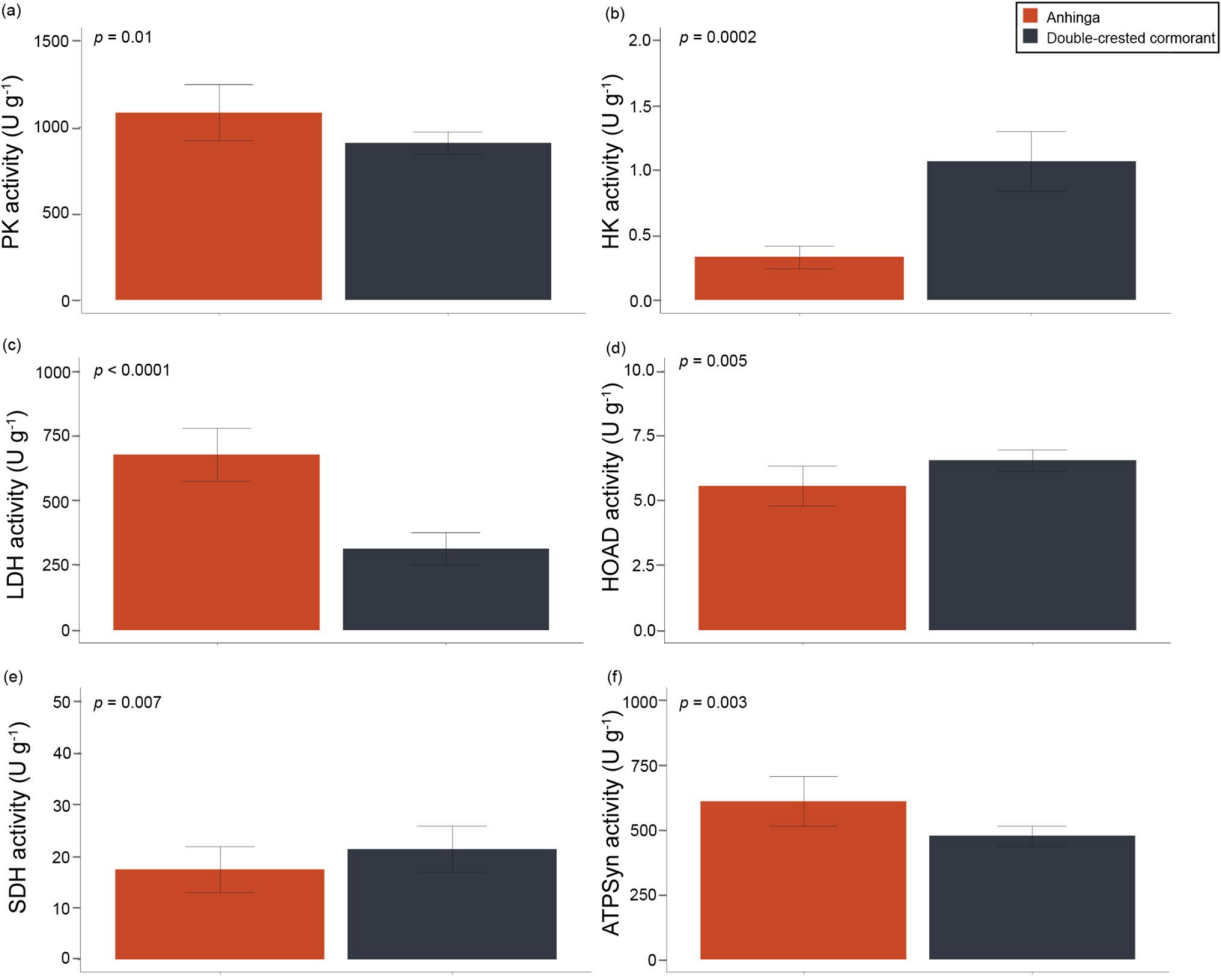
Maximal activity mean and standard deviation (SD) in the pectoralis for six mitochondrial enzymes, which showed significant differences between anhingas (*n* = 10) and double-crested cormorants (*n* = 10), including **a** pyruvate kinase (PK), **b** hexokinase (HK), **c** lactate dehydrogenase (LDH), **d** 3-hydroxy-acyl-CoA dehydrogenase (HOAD), **e** succinate dehydrogenase (SDH), and (f) ATP synthase (ATPSyn). Activity was measured as µmol g^–1^ tissue min^–1^ (U g^–1^). Significance levels were derived from t-tests or Kruskal-Wallis tests with the alpha value set as *p* < 0.05

**Fig. 4:**
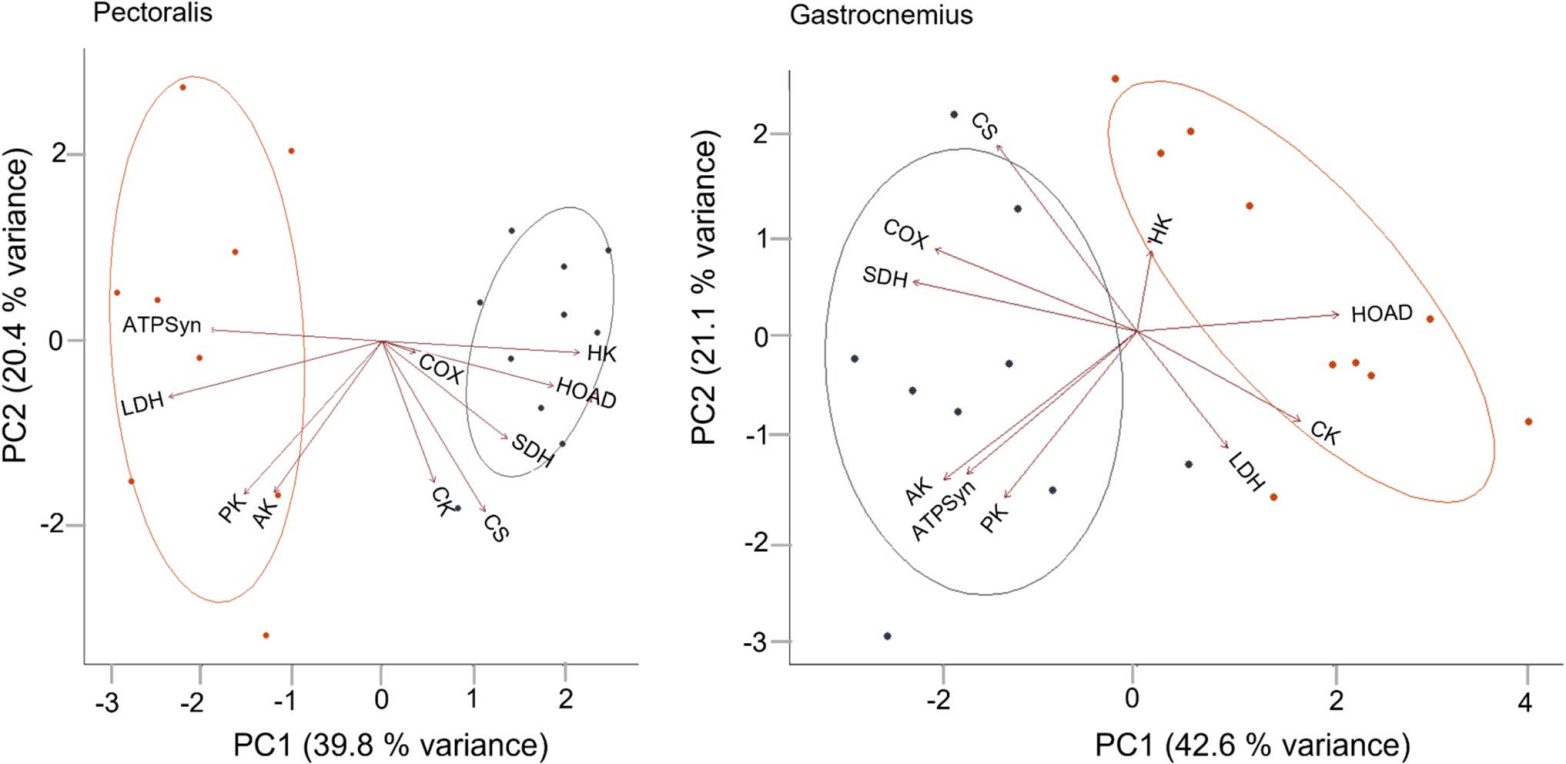
Principal components analysis (PCA) of the 10 enzymes assayed across all individual anhingas (*n* = 10; orange) and double-crested cormorants (*n* = 10; grey) in the pectoralis (right) and gastrocnemius (left). The two species were primarily separated along PC1, although the direction of the loading reversed between the muscles. Enzymes include hexokinase (HK), pyruvate kinase (PK), lactate dehydrogenase (LDH), 3-hydroxy-acyl-CoA dehydrogenase (HOAD), citrate synthase (CS), succinate dehydrogenase (SDH), cytochrome c oxidase (COX), ATP synthase (ATPSyn), creatine kinase (CK), and adenylate kinase (AK)

Our data show mixed results in the oxidative capacity of the pectoralis based on the maximal activity of the enzymes assayed (Fig. 3; Online Resource 3–6). The double-crested cormorant had significantly higher SDH (*p* = 0.0001), whereas the anhinga expressed higher ATPSyn activity (*p* = 0.003). Enzymes associated with glycolysis were similarly divided with the double-crested cormorant having higher HK (*p* = 0.0002) and the anhinga having higher PK (*p* = 0.01). The double-crested cormorant had a higher lipid oxidation capacity with significantly higher HOAD (*p =* 0.005), HOAD/HK (*p* = 0.0002), and ATPSyn/CS (*p* = 0.003). However, it also had a higher glycolytic potential (LDH/CS, *p* = 0.0002; PK/CS, *p* = 5.6 × 10^–5^). Overall, the pectoralis of the anhinga showed a higher capacity for carbohydrate metabolism (LDH, *p* = 4.0 × 10^–7^) than for lipids. In the PCA, PC1 explained 39.8% of the variation. The double-crested cormorant had positive loadings for HK, HOAD, and SDH, whereas the anhinga had negative loadings for ATPS, LDH, PK, and AK (Fig. 4).

### Enzyme activity in the gastrocnemius

**Fig. 5:**
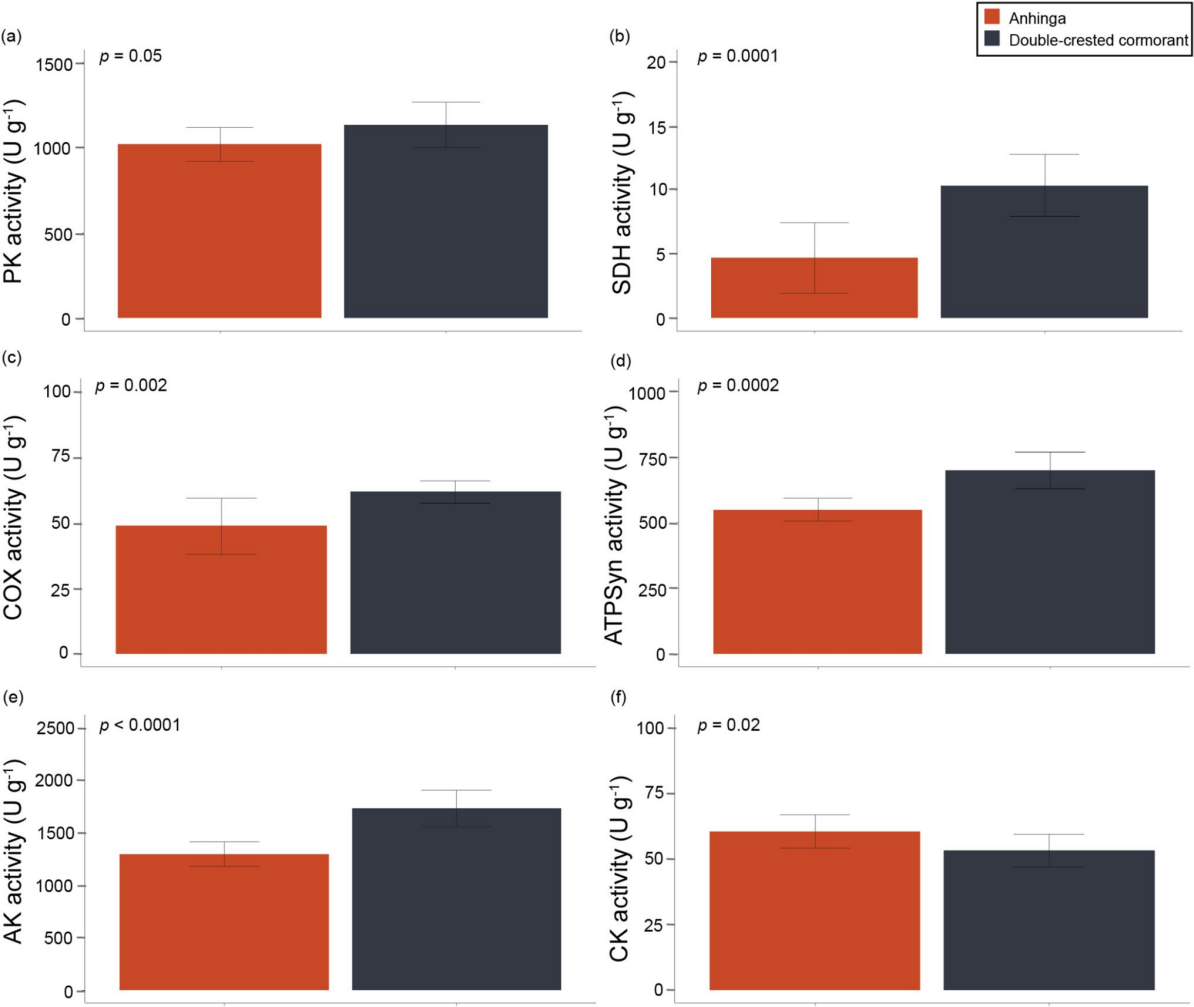
Maximal activity mean and standard deviation (SD) in the gastrocnemius for six mitochondrial enzymes that indicated significant differences between anhingas (*n* = 10) and double-crested cormorants (*n* = 10) including **a** pyruvate kinase (PK), **b** succinate dehydrogenase (SDH), **c** cytochrome c oxidase (COX), **d** ATP synthase (ATPSyn), **e** adenylate kinase (AK), and **f** creatine kinase (CK). Activity was measured as µmol g^–1^ tissue min^–1^ (U g^–1^). Significance levels were derived from t-tests or Kruskal-Wallis tests with the alpha value set as *p* < 0.05

Our results suggest that the double-crested cormorant had a higher oxidative capacity in the gastrocnemius than the anhinga, but both showed a predominance of glycolytic fibers (Fig. 5; Online Resource 3–5,7). Key enzymes associated with oxidative phosphorylation (COX, *p =* 0.002; SDH, *p* = 0.0001) and the production of ATP (ATPSyn, *p* = 4.0 × 10^–5^) were significantly higher in the double-crested cormorant. Additionally, AK was higher (*p* = 7.3 × 10^–6^) suggesting an elevated capacity for shuttling ATP and adenylate metabolism. These differences were also reflected in the PCA (Fig. 4). PC1 explained 42.6% of the variation. PC1 showed positive loadings of HOAD and HK, which separated the anhingas from the negative loadings of PK, ATPSyn, AK, SDH, and COX for the double-crested cormorant.

### Enzyme activity in the left ventricle

Finally, we assessed maximal enzyme activity in the left ventricle of the heart (Online Resources 3–5). The only significant differences between the double-crested cormorant and the anhinga in enzyme activity of the left ventricle were in HOAD (*p* = 1.8 × 10^–5^), ATPSyn (*p* = 0.0001), AK (*p* = 0.002), and CS (*p* = 0.02). Overall, these indicate that the double-crested cormorant left ventricle has a higher oxidative capacity and lipid metabolism than the anhinga.

### Blood- and muscle-O_2_ storage capacity

We then assessed blood-O_2_ storage capacity and found that anhingas and double-crested cormorants did not differ in [Hb], Hct, or MCHC (Table 2; Online Resource 2). Next, we measured [Mb] and found no significant differences between the two species in the gastrocnemius, pectoralis, or the left ventricle (Table 1; Online Resource 2). We did find a slight difference between the pectoralis and the left ventricle (*p* = 0.05) in the double-crested cormorant, suggesting a higher O_2_ storage potential in the pectoralis (Table 2).

**Table 2:** Mean and standard deviation (SD) of O_2_ storage parameters measured in anhingas (*n* = 10) and double-crested cormorants (*n* = 10). We found no significant differences between the two species in any parameter. Results of full statistical analyses are given in Online Resource 2. Significance levels were derived from t-tests or Kruskal-Wallis tests with the alpha value set as *p* < 0.05.

Trait	Anhinga	Double-crested cormorant
[Hb] (g dL^–1^)	14.4 ± 1.2	13.7 ± 2.0
Hct (%)	37.5 ± 3.7	39.1 ± 6.7
MCHC (g dL^–1^)	38.8 ± 2.1	31.7 ± 11.4
[Mb] (mg g^–1^)		
Gastrocnemius	5.6 ± 2.9	6.5 ± 2.1
Pectoralis	8.6 ± 4.0^4^	10.6 ± 2.1
Left ventricle	4.8 ± 1.1	5.9 ± 1.7

^4^Anhinga pectoralis Mb measured on *n *= 8 individuals

We next measured the mass of the gastrocnemius, pectoralis, heart, and lungs to determine their relative proportion to the total body mass (Table 1; Online Resource 2). The double-crested cormorant had proportionally larger lungs (~ 1.4x larger; *p* = 0.0006) than anhingas. However, the pectoralis of the anhinga was proportionally larger than that of the double-crested cormorant (~ 0.8x larger, *p* = 5.5 × 10^–7^). Perhaps most surprising was the large difference in the proportional size of the gastrocnemius between these two species. The gastrocnemius in the double-crested cormorant accounted for ~ 2.8x more of the total body mass than in the anhinga (*p* = 7.4 × 10^–5^). Whereas there was no significant difference in the [Mb] in the gastrocnemius, the difference in mass indicates that the double-crested cormorant should have more available Mb. We found that the amount of available Mb in the gastrocnemius was ~ 5x higher in the double-crested cormorant (214.0 ± 86.5 mg) than in the anhinga (40.5 ± 23.9 mg).

### Dive duration and surface time of the anhinga

We aimed to compare how differences in diving phenotype predict the average diving time for the anhinga and the double-crested cormorant. While there are several published measurements of double-crested cormorant dives (both free and forced) yielding an average dive time of ~ 20 s (Enstipp et al. 2006; Halsey et al. 2007), no published times for anhingas could be found. Therefore, we timed the foraging dives (*n* = 75) of anhingas (*n* = 11 individuals) freely diving in a suburban lake in southwest Florida. Dives averaged 24 ± 17 s and reached a maximum of 71 s. This is similar to what Ryan (2007) recorded for African darters (*Anhinga rufa*). Most dives occurred in very shallow water (< 1 m) within the emergent vegetation at the edges of the lake (mean 18 ± 11 s) and had an extremely high capture rate of small fish which were brought to the surface to swallow (> 90% of dives). Dives occurring in the middle of the lake were longer (mean = 42 ± 20 s; *p* = 1.5 × 10^–5^), but whether this is due to increased depth, horizontal distance, or low prey capture rate could not be determined. As the average dive duration for the anhinga was not statistically different from the double-crested cormorant, we were not able to assess the influence that any of our reported traits may have on dive duration. Surface times for anhingas similarly varied according to dive duration (*p* = 1.4 × 10^–5^) with shallow dives averaging 6 ± 3 s and mid-lake dives averaging 14 ± 4 s at the surface.

## Discussion

Overall, our results suggest that the diving phenotypes of the double-crested cormorant and the anhinga differ substantially. The double-crested cormorant has a more oxidative phenotype with higher reliance on aerobic metabolism to fuel energetically demanding foraging behavior underwater. This is likely fueled by an increased O_2_ reservoir associated with a larger quantity of Mb available in the gastrocnemius due to the proportionally larger size of that muscle in the double-crested cormorant. The anhinga, on the other hand, exhibits a unique diving phenotype characterized by a higher glycolytic capacity than many other diving birds. The uncommon foraging behavior and low metabolism of the anhinga reduces the energy demands and subsequent reliance on large O_2_ stores needed while foraging underwater.

### Muscle fiber composition and capillarity

In both species, the pectoralis was composed of > 97% oxidative fibers. This proportion is higher than in Atlantic puffins (Kovacs and Meyers 2000; Table 3) or high-altitude flying bar-headed geese (*Anser indicus*; Scott et al. 2009). It is, however, similar to deep diving waterfowl such as the long-tailed duck (*Clangula hymalis*; Schell et al. 2024b; Table 3). It is possible that these high oxidative proportions are related to their high wing-loading (Owre 1967). High wing-loading in volant divers has been linked to increased dive duration (Watanuki and Burger 1999; Halsey et al. 2007), flight speed (Rayner 1988), and decreased buoyancy (Lapsansky et al. 2022). Unlike species such as dabbling ducks, which are able to ‘rocket’ vertically from the water (Rosser and George 1986; Norberg 2012), cormorants require extensive takeoff periods to get airborne, as do anhingas. By contrast, these ‘rocketing’ species have a large glycolytic component to their pectoralis to accommodate this burst activity. Therefore, the fast, powered flight and extensive takeoff period of the double-crested cormorant and alternating powered-soaring flight of the anhinga would benefit from more oxidative fibers fueled by Mb-bound O_2_ to sustain aerobic flight without rapid fatigue. Further studies into the exercise physiology of their flight would be beneficial in determining the degree of functional differences these traits may impart.

Another possible factor necessitating this high oxidative percentage in both the anhinga and the double-crested cormorant is the shared behavior of wing-spreading. This behavior is unique to cormorants and darters and is a trade-off required by their wettable plumage. Holding the wings outstretched for extended periods requires modifications in the wing muscles to avoid fatigue (Henneman, 1983; Meyers 1997). A higher proportion of oxidative fibers in the pectoralis could contribute to sustaining wing-spreading posture without fatigue while the feathers dry.

The muscle fiber composition of the gastrocnemius in the double-crested cormorant is comparable to what is reported for dabbling ducks (Schell et al. 2024b; Table 3). However, the anhinga has a much higher glycolytic fiber composition suggesting a higher capacity for burst activity while underwater. The transverse area of the glycolytic fibers in both species is also large compared to ducks (Norberg 2012; Schell et al. 2024b; Table 3). This higher glycolytic fiber composition in the gastrocnemius of both species suggests a high capacity for burst activity. In both, this would be a benefit to prey capture, particularly in the overall less energetic foraging style of the anhinga. It would also provide a quick surge of energy to help propel the double-crested cormorant along the water’s surface during take-off.

The diffusion capacity of O_2_ into the tissues can be augmented by increasing the number and density of blood capillaries supplying the muscle (Leόn-Velarde et al. 1991; Torrella et al. 1998; Scott et al. 2009). Yet in diving mammals and penguins capillary density in the swimming muscles is lower than would be expected, indicating preferential reliance on intramuscular Mb-bound O_2_ stores rather than blood O_2_ (Ponganis et al. 1997a; Kooyman and Ponganis 1998; Kanatous et al. 1999). In both the anhinga and the double-crested cormorant, capillary density and the capillary to fiber ratio was lower than has been reported for the long-tailed duck but similar to shallow and non-diving duck species (Schell et al. 2024b; Table 3). Again, suggesting that blood-O_2_ may not be the primary O_2_ reservoir for these two species during dives.

**Table 3:** Comparison of traits associated with blood- and muscle-O_2_ storage and metabolism between anhingas and double-crested cormorants and other diving bird species. All anhinga and double-crested cormorant enzyme assays were performed concurrently with the duck samples from Schell et al. (2023). Overall, the double-crested cormorant exhibits a diving phenotype that is intermediate between deep diving ducks (i.e., long-tailed duck) and non-diving dabbling ducks (i.e., American wigeon). The anhinga shows a uniquely less oxidative phenotype that is more similar to non-diving species

Trait	Anhinga	Double-crested cormorant	Long-tailed duck	Lesser scaup	American wigeon	Emperor penguin	Atlantic puffin
	*Anhinga anhinga*	*Nanopterum auritum*	*Clangula hymealis*	*Aythya affinis*	*Mareca americana*	*Aptenodytes forsteri*	*Fratercula arctica*
References	A	A, B	C, D	C, E	C	F	G
Average dive duration (minutes)	< 1	< 1	< 1	< 0.5	0	< 5–10	< 1
Maximum dive duration (minutes)	1.2	1	1	< 0.5	0	27.6	1.9
*Gastrocnemius*							
Mass (g)	7.3	31.6	3.4	3.7	3.7	–	–
Percent total mass (%)	0.5	1.4	0.4	0.5	0.5	–	–
Glycolytic fiber areal density (%)	72.2	60.4	39.4	53.1	57.4	–	–
Oxidative fiber areal density (%)	27.8	39.6	60.6	47.0	42.6	–	–
Glycolytic fiber mitochondrial volume density	0.12	0.17	0.31	0.14	0.15	–	–
Subsarcolemmal mitochondrial volume density	0.03	0.05	0.12	0.06	0.04	–	–
Intermyofibrillar mitochondrial volume density	0.09	0.11	0.19	0.13	0.11	–	–
Proportion subsarcolemmal	0.26	0.32	0.39	0.29	0.27	–	–
Oxidative fiber mitochondrial volume density	0.29	0.33	0.41	0.34	0.29	–	–
Subsarcolemmal mitochondrial volume density	0.11	0.13	0.20	0.15	0.12	–	–
Intermyofibrillar mitochondrial volume density	0.18	0.21	0.22	0.19	0.17	–	–
Proportion subsarcolemmal	0.38	0.38	0.49	0.43	0.41	–	–
Glycolytic fiber transverse area (µm^2^)	2706.36	2465.57	1712.0	2036.0	2073.0	–	–
Oxidative fiber transverse area (µm^2^)	1389.09	1377.44	1078.0	1809.0	1251.0	–	–
Capillary density (mm^–2^)	738.35	905.52	1175.0	950.6	866.0	–	–
Capillary/fiber ratio	1.33	1.62	2.11	1.70	1.78	–	–
Maximal enzyme activity (U g^–1^)							
Hexokinase (HK)	1.63	1.45	1.07	1.42	1.26	–	–
Pyruvate kinase (PK)	1021.14	1134.45	847.86	1026.54	945.38	–	–
Lactate dehydrogenase (LDH)	433.66	423.90	294.27	400.11	358.8	–	–
Hydroxy-acyl-CoA dehydrogenase (HOAD)	5.92	4.42	5.79	5.45	5.47	–	–
Citrate synthase (CS)	42.54	46.95	93.01	79.62	57.05	–	–
Succinate dehydrogenase (SDH)	4.72	10.40	21.98	12.54	11.73 6	–	–
Cytochrome c oxidase (COX)	48.66	61.89	149.56	127.11	50.94	–	–
ATP Synthase (ATPSyn)	550.21	700.20	403.96	596.07	718.16	–	–
Creatine kinase (CK)	60.44	53.34	35.06	43.3	36.49	–	–
Adenylate kinase (AK)	1297.72	1728.99	602.17	765.38	1071.42	–	–
Myoglobin concentration [Mb] (mg g^–1^)	5.6	6.5	9.8	6.2	3.9	20	6
*Pectoralis*							
Mass (g)	165.4	227.4	124.8	113.6	132.7	–	–
Percent total mass (%)	11.8	9.9	15.0	14.0	17.0	–	–
Glycolytic fiber areal density (%)	2.7	0.0	0.6	18.0	28.1	0	0
Oxidative fiber areal density (%)	97.3	100.0	99.4	82.0	71.9	100	100
Maximal enzyme activity (U g^–1^)							
Hexokinase (HK)	0.33	1.07	0.24	0.30	0.51	–	1.15
Pyruvate kinase (PK)	1084.10	909.03	1039.91	1079	1064.43	–	–
Lactate dehydrogenase (LDH)	675.90	314.25	257.77	426.72	590.4	1165	–
Hydroxy-acyl-CoA dehydrogenase (HOAD)	5.56	6.54	8.38	8.92	8.36	–	–
Citrate synthase (CS)	136.60	151.62	165.05	169.72	128.89	38	–
Succinate dehydrogenase (SDH)	17.42	21.34	35.04	27.84	20.03	–	–
Cytochrome c oxidase (COX)	274.67	297.77	410.58	374.33	205.49	–	–
ATP Synthase (ATPSyn)	611.36	478.30	453.86	632.98	660.07	–	–
Creatine kinase (CK)	1171.43	971.32	874.1	18.12	1026.02	–	–
Adenylate kinase (AK)	34.75	37.38	11.26	901.56	28.8	–	–
Myoglobin concentration [Mb] (mg g^–1^)	8.6	10.6	8.0	6.2	5.5	64	13
Hemoglobin concentration [Hb] (g dL^–1^)	14.4	13.7	16.8	16	13.7	18	–
Hematocrit (%)	37.5	39.1	47.8	46.2	41.8	50	57.9

References: (A) this study; (B) Enstipp et al. 2006; Halsey et al. 2007; (C) Schell et al. 2023, 2024a, b; (D) Ingram and Salmon 1941; (E) Alexander and Hair 1979; Custer et al. 1996; (F) Kirkwood and Robertson 1997 and Kooyman and Kooyman 1995; Ponganis et al. 1997a, b; Rodary et al. 2000, Sato et al. 2011, Wienecke et al. 2007; (G) Davis and Gurderley 1990, Kovacs and Meyers 2000; Wanless et al. 1992

#### Mitochondrial volume density and arrangement

Aerobic respiration during breath-hold dives can be maintained by increasing the aerobic capacity of the muscles themselves (Hochachka 1992); however, the mechanisms involved are highly variable among taxa (Reed et al. 1994). For example, some species increase the proportion of oxidative muscle fibers, whereas others increase mitochondrial volume density (Turner and Butler 1988; Scott et al. 2009; Mahalingam et al. 2017). Increasing the mitochondrial content of the muscle may increase O_2_ flux in the locomotory muscles (Hoppeler et al. 1987; Torrella et al. 1998; Kanatous et al. 1999; Scott et al. 2009). This increases O_2_ flux and extraction efficiency from the blood even when Hb- and Mb-bound O_2_ stores are depleted. Many high-altitude and diving species have adapted to hypoxia or intermittent hypoxemia, respectively, by increasing the subsarcolemmal subfraction of mitochondria in the muscles (Scott et al. 2009; Mahalingam et al. 2017; McCracken et al. 2024; Schell et al. 2024b). O_2_ delivery into the cells can be enhanced by translocating mitochondria towards the cell membrane closer to the capillaries to overcome resistance to diffusion at the blood-muscle barrier (Honig 1991; Dawson et al. 2016). This has been shown as an adaptation in the muscles of many species exposed to both intermittent and chronic hypoxia (Scott et al. 2009; Mahalingam et al. 2017; Schell et al. 2024b). However, translocation of mitochondria has a trade-off in increasing the diffusion distance for ATP and other metabolites within the cell, which must be mitigated to maintain normal cell function (Kinsey et al. 2007). Our findings are more similar to what is documented for the low-altitude barnacle goose (*Branta leucopsis*) and pink-footed goose (*Anser brachyrhynchus*), which had a larger intermyofibrillar subfraction than the high-altitude bar-headed goose (Scott et al. 2009).

Although we did find a larger subsarcolemmal subfraction in double-crested cormorant glycolytic fibers as compared to the anhinga, the majority of mitochondria in both species were located in the intermyofibrillar subfraction. This pattern is more similar to pinnipeds that have a high intermyofibrillar subfraction of 70–90% (Kanatous et al. 1999). This allows quick access to the large Mb-bound O_2_ stores in the sarcoplasm of locomotory muscles (Davis et al. 2004). The dive response of pinnipeds involves significant bradycardia and vasoconstriction, which induces slight tissue hypoxia to mobilize Mb-bound O_2_ (Davis and Kanatous 1999; Davis and Williams 2012). Diving ducks, on the other hand, increase blood flow to the locomotory muscles and rely heavily on blood-O_2_ (Bevan and Butler 1992). Double-crested cormorants appear to fall between these two strategies. During a dive, the double-crested cormorant’s heart rate remains above the resting rate, which facilitates the delivery of any available blood-O_2_ to the tissues (Enstipp et al. 2001). Based on our results, the intermyofibrillar mitochondria subfraction in the anhinga and the double-crested cormorant is intermediate between pinnipeds and diving ducks. Therefore Mb-bound O_2_ is likely an important fuel for aerobic respiration in both species as blood-O_2_ decreases.

#### Enzyme activity in the locomotory muscles

Despite being composed of predominantly oxidative fibers, our data suggest that the pectoralis of both the anhinga and the double-crested cormorant has a high glycolytic capacity. This is similar to both diving and dabbling ducks (Schell et al. 2023), but far below deep diving species like emperor penguins (*Aptenodytes forsteri;* Ponganis et al. 1997a, 1997b; Table 3). PK activity was slightly higher in the anhinga, but there were stark differences in both HK and LDH activity. The anhinga LDH activity was twice what was measured for the double-crested cormorant, but similar to dabbling ducks (Schell et al. 2023; Table 3) and black-crowned night herons (*Nycticorax nycticorax*; Franson et al. 1985). However, the double-crested cormorant appears to have a higher capacity for anaerobic activity in the pectoralis. HK, which is active in the first step of glycolysis, was more than three-times higher in the double-crested cormorant and significantly higher than was reported for diving ducks (Schell et al. 2023; Table 3). The double-crested cormorant also had elevated LDH/CS and PK/CS ratios. This may be indicative of short burst capacity, which would provide the energy needed to generate lift during the long takeoff runs that cormorants require to exit the water. Anhingas, on the other hand, typically gain lift by leaping off branches or banks and make use of thermals for soaring and gliding between flapping intervals. Therefore, the anhinga likely does not require such short burst energy for flight but would benefit from this capability when using their wings to counteract struggling prey underwater (White, pers. obs.).

In OXPHOS, the double-crested cormorant exhibited a higher oxidative capacity in the gastrocnemius than the anhinga. There was a concurrent increase in SDH, COX, and ATPSyn activity as well as a higher SDH/CS ratio. This increase in COX would suggest a higher mitochondrial abundance in the double-crested cormorant gastrocnemius and increase in the aerobic capacity of the leg muscles while swimming, a similar trend is seen in diving ducks (Schell et al. 2023, 2024b). Interestingly, we did not find a significant difference in CS activity which is often concurrent with increases in mitochondrial abundance. The double-crested cormorant is a more active swimmer compared to the anhinga, so increasing the oxidative capacity of the leg would sustain swimming and foraging longer without fatigue. This higher oxidative capacity suggests that mitochondrial O_2_ supplied by Mb is critical for maintaining high levels of activity while diving.

Although the anhinga had similar [Mb] in the gastrocnemius, we found significantly lower oxidative capacity overall. In particular, SDH activity was much lower than expected in both the gastrocnemius and the pectoralis. SDH activity has been shown to be low in species that exhibit more sedentary behaviors (George and Talesara 1961) and high in those with high O_2_ demands (Torrella et al. 1998). Unlike cormorants or diving ducks, anhingas forage by slowly and methodically swimming through vegetation searching for concealed prey, sometimes lying in wait and then stabbing at prey, which is much less energetically demanding (Owre 1967; Hennemann 1983, 1985). Their rapid plumage saturation and unique morphology also allows the anhinga to be neutrally buoyant just below the water’s surface (Casler 1973), further decreasing energy demand under water. The related African darter uses minimal kicking of their feet to maintain their position underwater when foraging (Ryan 2007) further indicating the relative ease at which they remain submerged. These unique behaviors could explain the surprisingly low SDH activity and lower oxidative capacity overall in the anhinga.

Both species showed higher activity in enzymes associated with substrate level phosphorylation than ducks (Schell et al. 2023; Table 3). This is most evident in the activity of AK, which was more than double that reported for diving ducks. AK is a regulator of ATP production and utilization, but when coupled with elevated CK is a strong indicator of increased muscle burst power (Saks 2008; Banerjee and Chaturvedi 2016). This capacity for burst power would be consistent with the high proportion of glycolytic fibers present in the gastrocnemius of both species. The ability to rapidly activate the locomotory muscles underwater while foraging would provide a surge of energy in the legs to propel them forward to capture prey.

Unlike what has been reported for diving ducks (Schell et al. 2023), double-crested cormorants and anhingas had higher ATPSyn/CS ratios, particularly in the gastrocnemius. High ATPSyn/CS ratios coupled with increased ATPSyn activity overall could be indicative of the lack of significant translocation of mitochondria to the subsarcolemmal subfraction. The subsarcolemmal mitochondria typically have a lower ATPSyn activity than intermyofibrillar mitochondria (Dawson and Scott 2022). This high ATPSyn/CS ratio is consistent with a high intermyofibrillar subfraction of mitochondria in the gastrocnemius. This would allow for their preferential enrichment due to their proximity to the intermyofibrillar Mb-bound O_2_ stores.

### O_2_ storage capacity

Overall, we found no difference in blood- or muscle- O_2_ storage capacity between the double-crested cormorant and the anhinga. [Hb], Hct, and [Mb] are known to vary with season (Sergent et al. 2004; Fair et al. 2007), age (Ponganis et al. 1999), or exercise (Butler 1988) in other avian divers. However, our measured values for the double-crested cormorant Hct are similar to what has been previously reported for the species (Kuiken and Danesik 1999). Therefore, we propose that the values reported here are representative. As expected, all O_2_ storage parameters were consistently lower in both species than have been reported for deeper diving cormorants such as the imperial cormorant (*Phalacrocorax atriceps*; Gallo et al. 2013). However, both species also showed a lower capacity for blood-O_2_ storage than was recently reported for diving ducks (Schell et al. 2023; Table 3). [Hb] and Hct in both species were more similar to non-diving dabbling duck species (Schell et al. 2024a) and white pelicans (*Pelecanus onocrotalus;* Puerta et al. 1991). Given these low [Hb] and Hct values, it is unlikely that blood-O_2_ stores are the primary source of O_2_ while diving for either of these species.

Marine mammals and some seabirds tend to have very high [Mb] in their locomotory muscles to provide quick access to O_2_ while diving (Weber et al. 2007; Haggblom 1987; Kooyman and Ponganis 1998; Dolar et al. 1999; Mirceta et al. 2013). The relative sizes of Mb-rich muscles themselves may also be increased, which further increases the size of the O_2_ store (Noren and Williams 2000). This Mb may help facilitate O_2_ movement into the muscle through the translational diffusion of oxyMb further enhancing O_2_ availability during dives (Wittenberg 1970). However, for volant divers there is a trade-off between [Mb] and energy production (i.e., mitochondrial abundance), especially in the primary flight muscle the pectoralis (Scott et al. 2009; Elliot et al. 2010). [Mb] in the anhinga and double-crested cormorant pectoralis was lower than in other volant seabirds such as the Atlantic puffin and common murre (*Uria aalge*; Davis and Guderley 1990; Table 3). However, the puffin and murre are both wing-propelled divers, whereas the double-crested cormorant and anhinga are primarily leg-propelled. Interestingly, the Japanese cormorant (*Phalacrocorax capillatus*) has higher [Mb] in the gastrocnemius than the double-crested cormorant or the anhinga (Yamamoto et al. 2011). Notably, the [Mb] in the gastrocnemius of both the anhinga and the double-crested cormorant are significantly higher than what was reported for diving ducks (Schell et al. 2024a). This suggests that although [Mb] is not as high as some deep diving species, Mb-bound O_2_ stores are a significant source of O_2_ for both of these species. The comparatively low blood-O_2_ storage capacity in both species further emphasizes this probable reliance on O_2_ stored in the muscles.

From this, we would expect that the muscles themselves could be modified to capitalize on these Mb stores while diving. The proportionally larger gastrocnemius of the double-crested cormorant, as compared to the anhinga or diving ducks (Schell et al. 2024b), is likely key to their overall oxidative capacity. This equates to a large amount of Mb available in the muscle itself to store O_2_. The large reservoir of O_2_ present in the double-crested cormorant gastrocnemius thus supports a more oxidative phenotype, especially as Mb and mitochondria are colocalized and interactive (Yamada et al. 2013).

## Conclusion

This study explored differences in diving phenotypes between two iconic diving species in South Florida, the double-crested cormorant and the anhinga. Our results suggest that the double-crested cormorant has a higher oxidative capacity than the anhinga, but they showed less remodeling of blood O_2_ storage capacity, muscle composition, mitochondrial arrangement, and enzyme activity in the primary swimming muscle than expected. In general, the double-crested cormorant phenotype most closely resembles that of the mid-depth diving pochard ducks reported in Schell et al. (2023, 2024a, 2024b). This intermediate phenotype between deep divers and non-divers suggests that double-crested cormorant dives are highly oxidative, likely relying on Mb-bound O_2_ reserves in their large gastrocnemius. This is complemented by a capacity for anaerobic metabolism, which may fuel burst activity related to prey capture and takeoff from the water.

The anhinga was much more similar to the non-diving dabbling ducks, with a slightly higher capacity for glycolysis. Their unique morphology and fully saturable plumage allows them to be neutrally buoyant in ~ 1 to 4 m of water within seconds (Owre 1967; Casler 1973; Rijke et al. 1989). These physical adaptations, plus their slower than expected metabolism (Hennemann 1985), allow the anhinga to adopt a less energetically taxing forage style (Owre 1967; Casler 1973; Henneman 1985). Anhingas also do not rely on fast, powered flight, instead alternating between powered flaps and periods of gliding or extended soaring on thermals. Together, these behaviors and adaptations result in a more glycolytic phenotype than other divers, giving the anhinga an extremely unique diving strategy compared to the otherwise highly oxidative diving phenotypes common in other diving species.

## Supplementary Information

Below is the link to the electronic supplementary material.

Supplementary file1 (DOCX 454 KB)

## Abbreviations

[Hb]: Hemoglobin concentration
Hct: Hematocrit
MCHC: Mean corpuscular hemoglobin concentration
[Mb]: Myoglobin concentration
HK: Hexokinase
PK: Pyruvate kinase
LDH: Lactate dehydrogenase
HOAD: 3-hydroxy-acyl-CoA dehydrogenase
CS: Citrate synthase
SDH: Succinate dehydrogenase
COX: Cytochrome c oxidase
ATPSyn: ATP synthase
CK: Creatine kinase
AK: Adenylate kinase
OXPHOS: Oxidative phosphorylation
O_2_: Oxygen
