# Supplementary material for: Comparative mechanisms for O2 storage and metabolism in two Florida diving birds: the anhinga (Anhinga anhinga) and the double-crested cormorant (Nannopterum auritum)
Source: J Comp Physiol B. 2024 Dec 20;195(2):191–208. doi: 10.1007/s00360-024-01593-x (PMC12069429; doi:10.1007/s00360-024-01593-x)
Supplement: Supplementary file 1 — Supplementary file1 (DOCX 454 KB) [file 360_2024_1593_MOESM1_ESM.docx]

Supplemental Tables and Figures

| Online Resource 1. Summary of collection details for all anhingas and double-crested cormorants included in this study. Site codes: Smyrna Ready Mix (SRM) and Blackwater Creek Koi Farm (BWC). | | | | | | | | | | | |  |  |
| --- | --- | --- | --- | --- | --- | --- | --- | --- | --- | --- | --- | --- | --- |
|  |  |  |  |  |  |  |  |  |  |  |  |  | |
|  |  |  |  |  |  |  |  |  |  |  |  | |  |
| Species | ID | Sex | Time | Year | Date | Site | Latitude | Longitude | County | State |  | |  |
| Anhinga | ANHI 1 | Female | 17:30 | 2022 | 2-Feb | SRM | 26.79713 | –81.77463 | Charlotte | Florida |  | |  |
| Anhinga | ANHI 2 | Female | 12:43 | 2022 | 10-Feb | SRM | 26.79721 | –81.77453 | Charlotte | Florida |  | |  |
| Anhinga | ANHI 3 | Male | 15:40 | 2022 | 17-Feb | SRM | 26.7949 | –81.78347 | Charlotte | Florida |  | |  |
| Anhinga | ANHI 4 | Female | 17:40 | 2022 | 17-Feb | SRM | 26.7944 | –81.76884 | Charlotte | Florida |  | |  |
| Anhinga | ANHI 5 | Female | 18:30 | 2022 | 17-Feb | SRM | 26.79815 | –81.77388 | Charlotte | Florida |  | |  |
| Anhinga | ANHI 6 | Female | 11:56 | 2022 | 24-Feb | SRM | 26.79906 | –81.7916 | Charlotte | Florida |  | |  |
| Anhinga | ANHI 7 | Female | 18:52 | 2022 | 2-Mar | SRM | 26.79815 | –81.77388 | Charlotte | Florida |  | |  |
| Anhinga | ANHI 8 | Male | 13:40 | 2022 | 17-Mar | SRM | 26.79906 | –81.7916 | Charlotte | Florida |  | |  |
| Anhinga | ANHI 9 | Female | 15:49 | 2022 | 17-Mar | SRM | 26.79249 | –81.79209 | Charlotte | Florida |  | |  |
| Anhinga | ANHI 10 | Male | 17:56 | 2022 | 17-Mar | SRM | 26.79878 | –81.77455 | Charlotte | Florida |  | |  |
| Double-crested Cormorant | DCCO 1 | Female | 11:33 | 2021 | 12-Dec | BWC | 28.91626 | –81.4408 | Lee | Florida |  | |  |
| Double-crested Cormorant | DCCO 2 | Female | 13:45 | 2022 | 20-Jan | SRM | 26.794166 | –81.78113 | Charlotte | Florida |  | |  |
| Double-crested Cormorant | DCCO 3 | Female | 18:12 | 2022 | 20-Jan | SRM | 26.79777 | –81.77403 | Charlotte | Florida |  | |  |
| Double-crested Cormorant | DCCO 4 | Male | 14:38 | 2022 | 27-Jan | SRM | 26.79485 | –81.79097 | Charlotte | Florida |  | |  |
| Double-crested Cormorant | DCCO 5 | Male | 12:30 | 2022 | 2-Feb | SRM | 26.79489 | –81.78343 | Charlotte | Florida |  | |  |
| Double-crested Cormorant | DCCO 6 | Male | 18:06 | 2022 | 2-Feb | SRM | 26.79713 | –81.77463 | Charlotte | Florida |  | |  |
| Double-crested Cormorant | DCCO 7 | Male | 15:21 | 2022 | 10-Feb | SRM | 26.7946 | –81.7911 | Charlotte | Florida |  | |  |
| Double-crested Cormorant | DCCO 8 | Female | 18:23 | 2022 | 10-Feb | SRM | 26.79412 | –81.78103 | Charlotte | Florida |  | |  |
| Double-crested Cormorant | DCCO 9 | Male | 15:10 | 2022 | 24-Feb | SRM | 26.79489 | –81.78343 | Charlotte | Florida |  | |  |
| Double-crested Cormorant | DCCO 10 | Female | 17:18 | 2022 | 24-Feb | SRM | 26.7947 | –81.78087 | Charlotte | Florida |  | |  |
| Double-crested Cormorant | DCCO 11 | Male | 15:02 | 2022 | 10-Mar | SRM | 26.79496 | –81.78345 | Charlotte | Florida |  | |  |
| Double-crested Cormorant | DCCO 12 | Male | 19:14 | 2022 | 17-Mar | SRM | 26.79238 | –81.77937 | Charlotte | Florida |  | |  |

| Online Resource 2. Full statistical table of Welch's two sample t-tests and Kruskal-Wallis test results on blood- and muscle O_2_ storage, organ mass proportions, fiber types, and mitochondrial arrangement. | | | | |
| --- | --- | --- | --- | --- |
|  |  |  |  |  |
|  | *N* (individuals) | Degrees of freedom | Test statistic | *P*-value |
| [Hb] | 21 | 1 | *H* = 0.37 | 0.54 |
| Hct | 22 | 13.45 | *t* = –0.62 | 0.55 |
| MCHC | 21 | 9.53 | *t* = 1.85 | 0.1 |
| [Mb] |  |  |  |  |
| --Gastrocnemius | 20 | 16.57 | *t* = –0.8 | 0.43 |
| --Pectoralis | 18 | 15.99 | *t* = –0.94 | 0.36 |
| --Left ventricle | 20 | 17.1 | *t* = –1.35 | 0.19 |
| Body mass | 22 | 12.77 | *t* = –6.45 | 2.4 x 10^–5^ |
| *% of total mass* |  |  |  |  |
| --Gastrocnemius | 21 | 1 | *H* = 15.70 | 7.4 x 10^–5^ |
| --Pectoralis | 22 | 20 | *t* = 7.22 | 5.5 x 10^–7^ |
| --Heart | 22 | 15.07 | *t* = –1.6 | 0.13 |
| --Lungs | 20 | 15.82 | *t* = –4.3 | 0.0006 |
| *Gastrocnemius* | *.* |  |  |  |
| Number of fibers | 12 | 1 | *H =* 0.01 | 0.91 |
| Capillaries | 12 | 1 | *H =* 6.70 | 0.01 |
| Capillary**:**Fiber | 12 | 1 | *H =* 7.03 | 0.008 |
| Capillary density | 12 | 1 | *H =* 6.08 | 0.01 |
| *Glycolytic fibers* |  |  |  |  |
| --Glycolytic fiber transverse area | 20 | 9.81 | *t* = 0.69 | 0.51 |
| --Glycolytic areal density | 20 | 163.54 | *t* = –9.16 | 2.2 x 10^–16^ |
| --Total mitochondria | 12 | 8.19 | *t* = –2.38 | 0.04 |
| --Subsarcolemmal mitochondria | 12 | 8.63 | *t* = –2.71 | 0.02 |
| --Intermyofibrilar mitochondria | 12 | 8.2 | *t* = –1.91 | 0.08 |
| --Proportion subsarcolemmal | 12 | 10 | *t* = –3.36 | 0.007 |
| *Oxidative fibers* |  |  |  |  |
| --Oxidative fiber transverse area | 20 | 8.56 | *t* = 0.05 | 0.96 |
| --Oxidative areal density | 20 | 163.54 | *t* = –9.16 | 2.2 x 10^–16^ |
| --Total mitochondria | 12 | 6.38 | *t* = –1.5 | 0.18 |
| --Subsarcolemmal mitochondria | 12 | 5.7 | *t* = –0.85 | 0.43 |
| --Intermyofibrilar mitochondria | 12 | 9.55 | *t* = –2.16 | 0.06 |
| --Proportion subsarcolemmal | 12 | 7.12 | *t* = –0.17 | 0.87 |
| *Pectoralis* |  |  |  |  |
| --Oxidative areal density | 22 | 99.98 | *t* = –11.02 | 2.2 x 10^–16^ |
| --Glycolytic areal density | 22 | 99.98 | *t* = –11.02 | 2.2 x 10^–16^ |

Online Resource 3. Maximal activity of 10 enzymes in the anhinga and double-crested cormorant gastrocnemius, pectoralis, and left ventricle. Activity was measured as μmol g^–1^ tissue min^–1^ (U g^–1^). Enzymes include hexokinase (HK), pyruvate kinase (PK), lactate dehydrogenase (LDH), 3-hydroxy-acyl-CoA dehydrogenase (HOAD), citrate synthase (CS), succinate dehydrogenase (SDH), cytochrome c oxidase (COX), ATP synthase (ATPSyn), creatine kinase (CK), and adenylate kinase (AK). Significances were derived from t-tests or Kruskal-Wallis tests followed by Bonferroni post-hoc corrections with the alpha value set as *p* < 0.05 (**p* < 0.05, ***p* < 0.001; ****p* < 0.0001).

|  | *pectoralis* | | *gastrocnemius* | | *left ventricle* | |
| --- | --- | --- | --- | --- | --- | --- |
|  | Anhinga | Double-crested Cormorant | Anhinga | Double-crested Cormorant | Anhinga | Double-crested Cormorant |
| *glycolysis and lactate production* | |  |  |  |  |  |
| HK | 0.33 ± 0.09 | 1.07 ± 0.23^***^ | 1.63 ± 0.39 | 1.45 ± 0.37 | 3.55 ± 0.46 | 3.35 ± 0.47 |
| PK | 1084.10 ± 163.19^*^ | 909.03 ± 65.16 | 1021.14 ± 99.57 | 1134.45 ± 133.31^*^ | 949.21 ± 115.24 | 903.50 ± 70.05 |
| LDH | 675.90 ± 100.98^***^ | 314.25 ± 62.55 | 433.66 ± 31.53 | 423.90 ± 40.68 | 221.66 ± 14.71 | 215.80 ± 21.89 |
| *beta-oxidation* | |  |  |  |  |  |
| HOAD | 5.56 ± 95.18 | 6.54 ± 38.08^**^ | 5.92 ± 1.63 | 4.42 ± 0.43 | 4.59 ± 0.37 | 5.66 ± 0.45^***^ |
| *citric acid cycle* | |  |  |  |  |  |
| CS | 136.60 ± 20.96 | 151.62 ± 14.17 | 42.54 ± 12.29 | 46.95 ± 12.99 | 115.58 ± 10.68 | 126.58 ± 6.98^*^ |
| *electron transport chain* | |  |  |  |  |  |
| SDH | 17.42 ± 4.43 | 21.34 ± 4.45^**^ | 4.72 ± 2.75 | 10.40 ± 2.44^***^ | 6.80 ± 4.91 | 26.22 ± 4.89 |
| COX | 274.67 ± 121.06 | 297.77 ± 144.22 | 48.66 ± 10.86 | 61.89 ± 4.28^**^ | 181.49 ± 48.41 | 186.56 ± 46.30 |
| ATPSyn | 611.36 ± 95.18^**^ | 478.30 ± 38.08 | 550.21 ± 45.03 | 700.20 ± 69.78^***^ | 131.86 ± 51.91 | 229.56 ± 32.63^***^ |
| *substrate-level phosphorylation* | |  |  |  |  |  |
| CK | 1171.43 ± 10.77 | 971.32 ± 4.40 | 60.44 ± 116.66* | 53.34 ± 172.82 | 19.72 ± 3.34 | 15.71 ± 5.18 |
| AK | 34.75 ± 214.79 | 37.38 ± 332.11 | 1297.72 ± 6.34 | 1728.99 ± 6.21^***^ | 315.64 ± 81.51 | 434.89 ± 59.16^**^ |

Online Resource 4. Enzyme activity ratios in the anhinga and double-crested cormorant gastrocnemius, pectoralis, and left ventricle. Significances were derived from t-tests or Kruskal-Wallis tests followed by Bonferroni post-hoc correction with the alpha value set as *p* < 0.05 (**p* < 0.05, ***p* < 0.001; ****p* < 0.0001).

|  | Anhinga | | | Double-crested Cormorant | | |
| --- | --- | --- | --- | --- | --- | --- |
|  | *gastrocnemius* | *pectoralis* | *left ventricle* | *gastrocnemius* | *pectoralis* | *left ventricle* |
| CS**:**HOAD | 8.00 ± 3.67 | 24.72 ± 3.20 | 25.26 ± 2.49^*^ | 10.76 ± 3.18 | 23.27 ± 2.74 | 22.43 ± 1.35 |
| LDH**:**CS | 11.1 ± 3.48 | 5.02 ± 0.85^**^ | 1.93 ± 0.16^*^ | 9.89 ± 3.73 | 2.06 ± 0.31 | 1.71 ± 0.16 |
| PK**:**CS | 25.86 ± 7.40 | 7.98 ± 0.86^***^ | 8.25 ± 1.08^*^ | 26.18 ± 1.18 | 6.03 ± 0.59 | 7.15 ± 0.65 |
| PK**:**LDH | 2.36 ± 0.22 | 1.63 ± 0.30 | 4.29 ± 0.48 | 2.70 ± 0.46^*^ | 2.97 ± 0.48^***^ | 4.21 ± 0.33 |
| SDH**:**CS | 0.11 ± 0.04 | 0.15 ± 0.03 | 0.23 ± 0.06 | 0.18 ± 0.03^***^ | 0.23 ± 0.05^*^ | 0.21 ± 0.04 |
| COX**:**CS | 1.18 ± 0.24 | 2.08 ± 1.00 | 1.56 ± 0.33 | 1.42 ± 0.43 | 1.95 ± 0.89 | 1.48 ± 0.40 |
| ATPSyn**:**CS | 13.97 ± 4.28 | 4.57 ± 0.92^*^ | 1.16 ± 0.50 | 16.39 ± 6.08 | 3.17 ± 0.31 | 1.82 ± 0.26^*^ |
| HOAD**:**HK | 3.81 ± 1.33 | 18.07 ± 5.77^***^ | 1.32 ± 0.23 | 3.28 ± 1.18 | 6.40 ± 1.49 | 1.71 ± 0.25^*^ |

| Online Resource 5. Full statistical table of Welch's two sample t-tests and Kruskal-Wallis test results for maximal enzyme activity and key enzyme ratios of hexokinase (HK), pyruvate kinase (PK), lactate dehydrogenase (LDH), 3-hydroxy-acyl CoA dehydrogenase (HOAD), citrate synthase (CS), succinate dehydrogenase (SDH), cytochrome c oxidase (COX), ATP synthase (ATPSyn), creatine kinase (CK), and adenylate kinase (AK). | | | | | | | | | | |  |
| --- | --- | --- | --- | --- | --- | --- | --- | --- | --- | --- | --- |
|  |  |  |  |  |  |  |  |  |  |  |  |
|  |  |  |  |  |  |  |  |  |  |  |  |
|  |  | Gastrocnemius | | | Pectoralis | | | Left Ventricle | | |  |
|  |  |  | | |  | | |  | | |  |
|  | *N* (individuals) | Degrees of freedom | Test statistic | *P*-value | Degrees of freedom | Test statistic | *P*-value | Degrees of freedom | Test statistic | *P*-value |  |
| HK | 19 | 17.93 | *t* = 1.04 | 0.31 | 1.00 | *H* = 13.5 | 0.0002 | 18.00 | *t* = 0.96 | 0.35 |  |
| PK | 19 | 16.66 | *t* = –2.15 | 0.05 | 10.27 | *t* = 3.01 | 0.01 | 14.85 | *t* = 1.07 | 0.30 |  |
| LDH | 19 | 1.00 | *H* = 0.37 | 0.55 | 13.09 | *t* = 9.26 | 4.0 x 10^–7^ | 15.75 | *t* = 0.7 | 0.49 |  |
| HOAD | 19 | 1.00 | *H* = 6.61 | 0.01 | 11.75 | *t* = –3.45 | 0.005 | 17.31 | *t* = –5.84 | 1.8 x 10^–5^ |  |
| CS | 19 | 1.00 | *H* = 0.69 | 0.41 | 13.85 | *t* = –1.81 | 0.09 | 15.51 | *t* = –2.73 | 0.02 |  |
| SDH | 19 | 17.75 | *t* = –4.89 | 0.0001 | 16.81 | *t* = –3.08 | 0.007 | 18.00 | *t* = 0.27 | 0.79 |  |
| COX | 19 | 1.00 | *H* = 9.61 | 0.002 | 16.93 | *t* = –0.38 | 0.71 | 17.97 | *t* = –0.24 | 0.81 |  |
| ATPSyn | 19 | 15.39 | *t* = –5.71 | 4.0 x 10^–5^ | 10.28 | *t* = 3.92 | 0.003 | 15.15 | *t* = –5.04 | 0.0001 |  |
| CK | 19 | 17.99 | *t* = 2.53 | 0.02 | 10.38 | *t* = –0.68 | 0.51 | 1.00 | *H* = 3.57 | 0.06 |  |
| AK | 19 | 15.79 | *t* = –6.54 | 7.3 x 10^–6^ | 1.00 | *H* = 1.71 | 0.19 | 16.42 | *t* = –3.74 | 0.002 |  |
| CS**:**HOAD | 19 | 17.64 | *t* = –1.80 | 0.09 | 15.88 | *t* = 1.06 | 0.31 | 13.91 | *t* = 3.16 | 0.007 |  |
| LDH**:**CS | 19 | 17.92 | *t* = 0.75 | 0.46 | 1.00 | *H* = 13.5 | 0.0002 | 17.96 | *t* = 3.10 | 0.006 |  |
| PK**:**CS | 19 | 17.53 | *t* = –0.09 | 0.93 | 13.90 | *t* = 5.70 | 5.6 x 10^–5^ | 14.79 | *t* = 2.75 | 0.02 |  |
| PK**:**LDH | 19 | 1.00 | *H* = 3.86 | 0.05 | 15.23 | *t* = –7.49 | 1.8 x 10^–6^ | 15.88 | *t* = 0.44 | 0.67 |  |
| SDH**:**CS | 19 | 17.55 | *t* = –5.90 | 1.52 x 10^–5^ | 16.66 | *t* = –2.10 | 0.05 | 1.00 | *H* = 1.12 | 0.29 |  |
| COX**:**CS | 19 | 1.00 | *H* = 1.85 | 0.17 | 16.23 | *t* = 0.31 | 0.76 | 17.44 | *t* = 0.48 | 0.64 |  |
| ATPSyn**:**CS | 19 | 16.16 | *t* = –1.03 | 0.32 | 1.00 | *H* = 8.64 | 0.003 | 13.65 | *t* = –3.72 | 0.002 |  |
| HOAD**:**HK | 19 | 17.75 | *t* = 0.94 | 0.36 | 1.00 | *H* = 13.5 | 0.0002 | 17.83 | *t* = –3.69 | 0.002 |  |


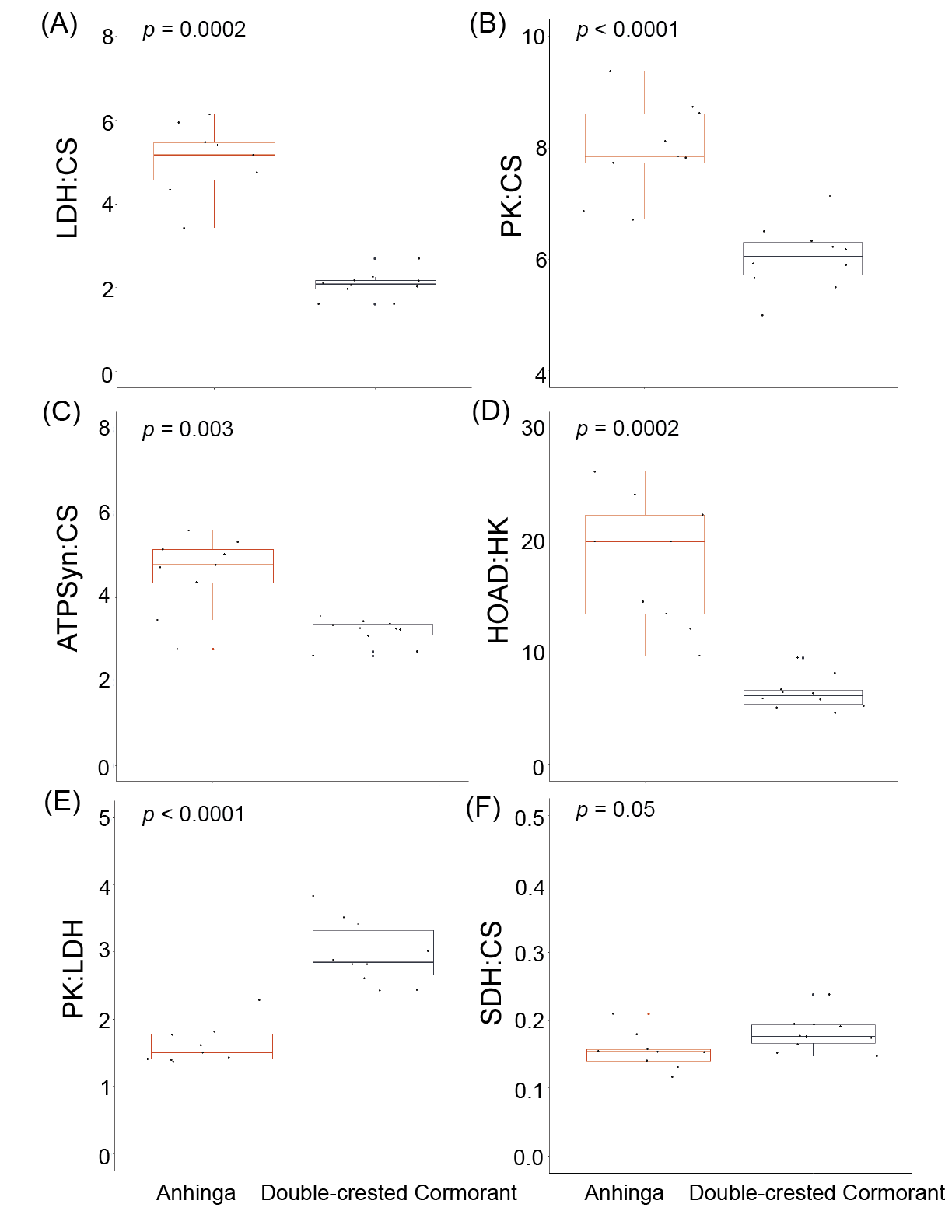


Online Resource 6. Enzyme ratios which indicated significant differences in the pectoralis between anhingas and double-crested cormorants including (a) LDH/CS, (b) PK/CS, (c) ATPSyn/CS, (d) HOAD/HK, (e) PK/LDH, and (f) SDH/CS. Boxplots show the median and quartile ranges of the data. Significances were derived from t-tests or Kruskal-Wallis tests by Bonferroni post-hoc tests with the alpha value set as *p* < 0.05.


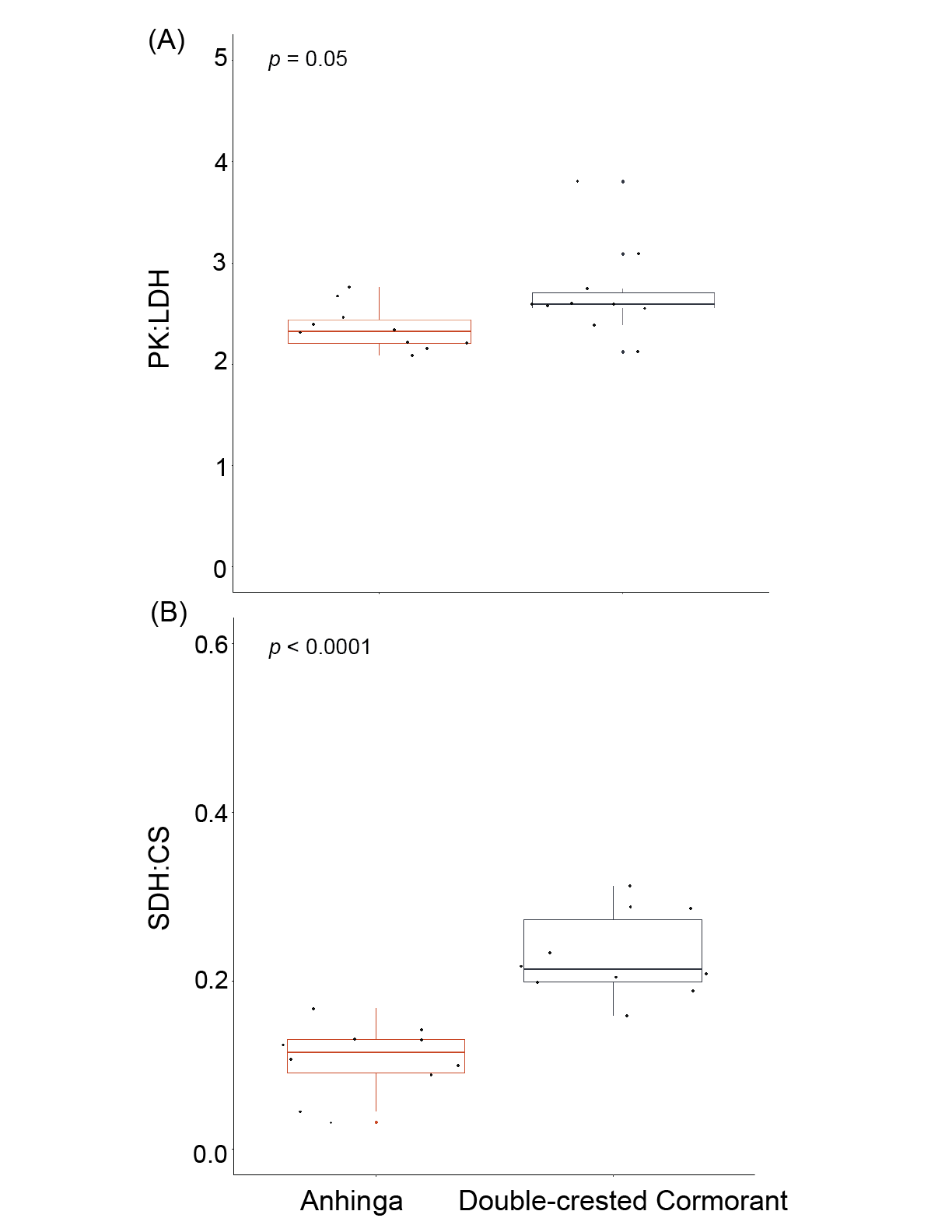


Online Resource 7. Enzyme ratios which indicated significant differences in the gastrocnemius between anhingas and double-crested cormorants including (a) PK/LDH, (b) SDH/CS. Boxplots show the median and quartile ranges of the data. Significances were derived from t-tests or Kruskal-Wallis tests followed by Bonferroni post-hoc tests with the alpha value set as *p* < 0.05.
